# Ethnopharmacology and Therapeutic Value of *Bridelia micrantha* (Hochst.) Baill. in Tropical Africa: A Comprehensive Review

**DOI:** 10.3390/molecules22091493

**Published:** 2017-09-08

**Authors:** Alfred Maroyi

**Affiliations:** Medicinal Plants and Economic Development (MPED) Research Centre, Department of Botany, University of Fort Hare, Private Bag X1314, Alice 5700, South Africa; amaroyi@ufh.ac.za; Tel.: +27-71-960-0326

**Keywords:** Africa, *Bridelia micrantha*, ethnopharmacology, herbal medicine, traditional uses

## Abstract

*Bridelia micrantha* is traditionally used in tropical Africa to treat a wide range of human and animal diseases. The aim of this study was to summarise the research that has been done on the ethnomedicinal uses, phytochemistry and pharmacological properties of *B. micrantha* so as to understand its importance and potential value in primary healthcare systems. The literature search for information on ethnomedicinal uses and pharmacological activities of *B. micrantha* was undertaken using databases such as Web of Science, Scopus, Google Scholar, Science Direct, BioMed Central (BMC), PubMed and Springerlink. Other relevant literature sources included books, book chapters, websites, theses, conference papers and other scientific publications. This study showed that *B. micrantha* is used as herbal medicine in just over half (57.3%) of the countries in tropical Africa where it is indigenous. A total of 54 ethnomedicinal uses of *B. micrantha* have been recorded with a high degree of consensus on burns, wounds, conjunctivitis, painful eyes, constipation, gastric ulcers, cough, headache, rheumatism, painful joints, dysentery, ethnoveterinary medicine, malaria, sexually transmitted infections, stomach ache, tape worms and diarrhoea. Different plant parts, aqueous and organic extracts exhibited anthelmintic, antimicrobial, anticonvulsant and sedative, antidiabetic, antidiarrhoeal, antinociceptive, antioxidant, antiplasmodial, antischistosomal, hepatoprotective, insecticidal and β-lactamase inhibitory activities.

## 1. Introduction

*Bridelia micrantha* (Hochst.) Baill. is a small to medium sized tree belonging to the family Phyllanthaceae (formerly Euphorbiaceae), commonly known as mitzeerie or coastal golden leaf [1]. The genus name “*Bridelia*” was coined in honour of Samuel Elisée Bridel-Brideri (1761–1828), a Swiss-German muscologist [2]. The species name, “*micrantha*” means “small-flowered” [2], in reference to the species’ very small flowers in auxillary clusters [3]. The genus *Bridelia* includes approximately 60–70 species found throughout tropical and subtropical regions of the world, particularly Africa and Asia [3,4]. Several *Bridelia* species are used in traditional medicine throughout the world as an anthelmintic, antiamebic, antianemic, antibacterial, anticonvulsant, antidiabetic, antidiarrhoeal, antiinflammatory, antimalarial, antinociceptive, antiviral, hypoglycemic and for abdominal pain, cardiovascular, gynecological and sexual diseases [4]. Thus it is not surprising that the bark, leaves and roots of *B. micrantha* are widely used as herbal medicines in tropical Africa [1], while the round and black berries of the species are widely eaten, particularly by children and can be used to make jams and juices [5,6]. *B. micrantha* has been identified as one of the few plant species that should be integrated in the domestication process in farming systems in sub-Saharan Africa to support medicinal, nutritional and income security of local communities through household use and marketing of its fresh or dried fruits [7]. At present, *B. micrantha* is domesticated as a fruit tree in Malawi [8] and as a medicinal tree in Tanzania [9]. Due to its popularity as a herbal medicine, *B. micrantha* is sold as such in the herbal medicine or “*muthi*” markets in Cameroon [10], Malawi [11], Nigeria [12] and South Africa [13]. *B. micrantha* is also an important timber tree species in tropical Africa, and the species is being overexploited as a source of wood for construction, poles, furniture, mortars, spoons and tool handles in Ethiopia [1,14] and Kenya [1]. The present review is aimed at documenting the ethnomedicinal uses, biological activities and the correlated chemical compounds of *B. micrantha* with emphasis on the validation of the ethnopharmacological uses of the species. Results of this review are expected to reveal research challenges and perspectives required to address the knowledge gaps of this important medicinal plant species in tropical Africa.

## 2. Research Methodology

*B. micrantha* and other historical names and synonyms of the species such as *Bridelia abyssinica* Pax, *Bridelia abyssinica* Pax var. *densiflora* Gehrm., *Bridelia mildebraedii* Gehrm., *Bridelia speciosa* Mϋell. Arg. var. *trichoclada* Mϋell. Arg., *Bridelia stenocarpa* Mϋell. Arg., *Bridelia zanzibarensis* Vatke & Pax, *Candelabria micrantha* Hochst. as well as common names such as “Benin ironwood”, “coastal golden leaf”, “mitzeerie” and “Yoruba ironwood” were used as the keywords in searching the major databases including Web of Science, Scopus, Google Scholar, Science Direct, BioMed Central (BMC), PubMed and Springerlink documenting traditional uses, medicinal uses, ethnobotany, ethnomedicinal uses, ethnopharmacology, pharmacology, phytochemistry and therapeutic value of the species. Additional literature, including pre-electronic literature such as scientific articles, conference papers, books, book chapters, dissertations, these and other grey material were sourced from the University of Fort Hare library in South Africa.

## 3. Occurrence, Distribution and Botanical Description

**Figure 1:**
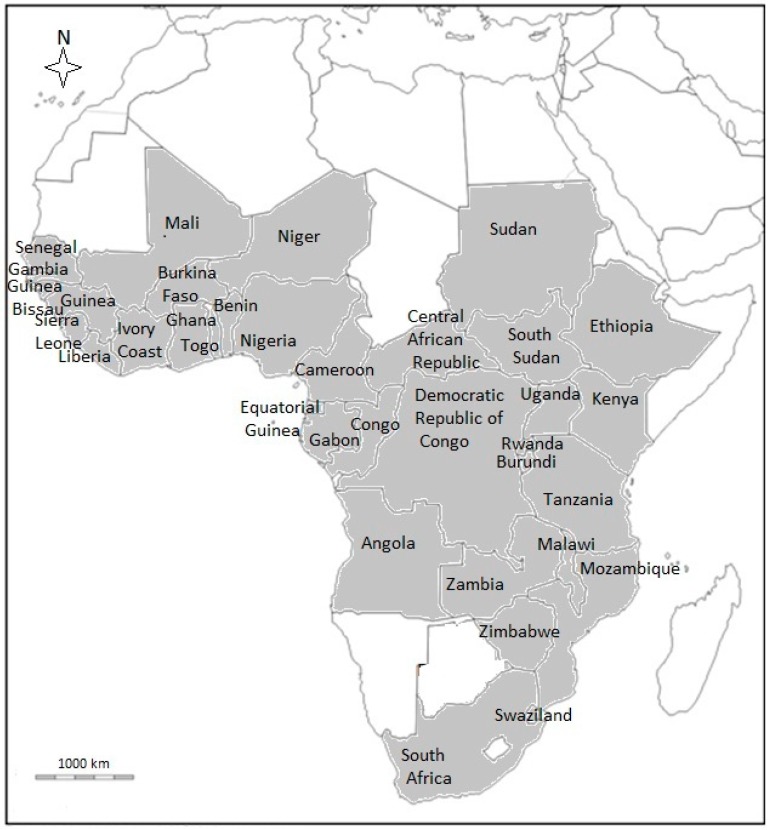
Distribution of *B. micrantha* in the mainland tropical Africa.

*B. micrantha* has been recorded in several countries throughout tropical Africa (Figure 1). The species has also been introduced on Réunion Island as a medicinal plant and is now naturalized on the island [1]. *B. micrantha* is found in a variety of habitats, ranging from savanna and woodland to seasonally flooded grassland, riverine forest, swamp forest and the margins of mangrove swamps, often from sea-level in West Africa to around 2500 m altitude in East Africa [1]. *B. micrantha* is a pioneer species that tolerates a wide diversity of soils, different rainfall regimes and can withstand moderate frost.

*B. micrantha* is an evergreen or deciduous, monoecious small to medium-sized tree up to 27 m tall with a short, often twisted bole up to 100 cm in diameter and a rounded crown [3]. The bark of *B. micrantha* is silver-grey to black in colour, smooth or rough with lenticels and reticulately fissured and flaking [2,3]. Young branches of *B. micrantha* often have spines and occasionally blunt spines are found on older branches. Leaves are simple, entire, distichous, often alternate, glabrous to slightly hairy, elliptical to oblong in shape. Flowers occur in clusters in leaf axils, yellow in colour, unisexual with triangular sepals and small petals [2,3]. Male flowers have stamens and filaments that are fused into a column at the base, but free and spreading above with rudimentary ovary. Female flowers are nearly sessile with ovary and styles fused at the base. The fruit is a globose, fleshy drupe, black in colour when ripe with brownish seeds [3].

## 4. Ethnomedicinal Uses of *B. micrantha*

The bark, leaf sap, leaves and roots of *B. micrantha* are reported to possess diverse medicinal properties and cure various human ailments and diseases throughout its distribution range in tropical Africa. A total of 54 ethnomedicinal uses of *B. micrantha* have been recorded (Table 1).

**Figure 2:**
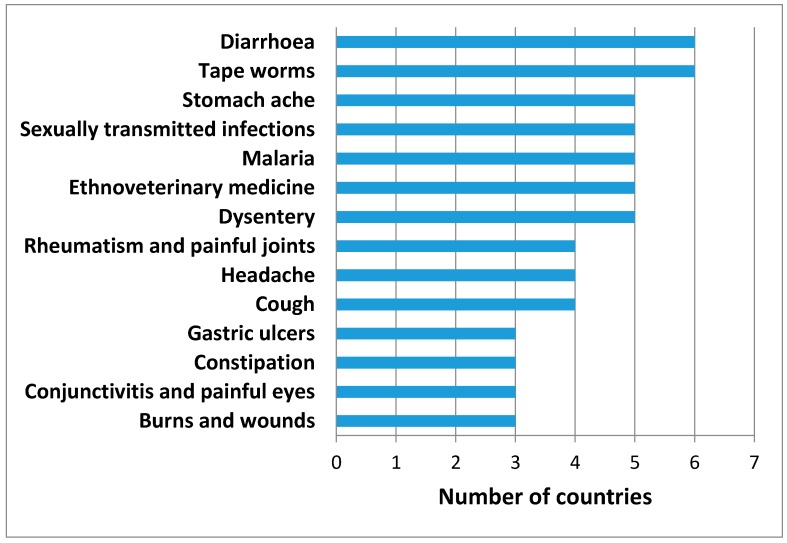
Cross-Cultural agreement among ethnomedicinal uses of *B. micrantha* in three or more countries in the tropics.

There is cross-cultural agreement among ethnomedicinal uses of *B. micrantha* with a high degree of consensus on burns and wounds, conjunctivitis and painful eyes, constipation, gastric ulcers, cough, headache, rheumatism and painful joints, dysentery, ethnoveterinary medicine, malaria, sexually transmitted infections (STIs), stomach ache, tape worms and diarrhoea (Figure 2). Ethnomedicinal information has been found in Burkina Faso, Burundi, Cameroon, Democratic Republic of Congo (DRC), Ethiopia, Gambia, Ghana, Guinea, Guinea-Bissau, Ivory Coast, Kenya, Nigeria, Sierra Leone, South Africa, Tanzania, Uganda and Zimbabwe, representing 57.3% of the countries where *B. micrantha* is indigenous. The country with the highest ethnomedicinal uses is South Africa (20 based on 12 literature records), followed by Cameroon with 18 uses and six literature records, DRC with 14 uses and three literature records, Tanzania with 12 uses and five literature records, Kenya with 11 uses and six literature records, and Nigeria with nine uses and seven literature records.

## 5. Phytochemical Constituents of *B. micrantha*

Multiple classes of phytochemicals including alkaloids, anthocyanidin, anthraquinones, carbohydrates, cyanogenic glycoside, essential oil, ester, flavonoids, oxalate, phenolic compounds, saponins, sterols, tannins, terpenoids as well as several minerals have been isolated from the bark, fruits, leaves and roots of *B. micrantha* [4,36,46,69,70,71,72,73,74,75,76,77]. Bark, fruits and leaves of *B. micrantha* contain a wide variety of classic nutrients, such as minerals, carbohydrates, polyol (hexahydroxy alcohol), and proteins (Table 2). Several chemical elements such as calcium, chromium, cobalt, copper, iron, lead, magnesium, manganese, nickel, phosphorus, potassium, sodium and zinc have been isolated from *B. micrantha* fruits (Table 2). Shelembe [46] found that the concentrations of elements in the fruits of *B. micrantha* were in the decreasing order of Mg > Ca > Mn > Zn > Fe > Cu > Co > Ni ≈ Cr > Pb. The dietary reference intake (DRI) of some of the chemical elements is shown in Table 2 based on the FAO or WHO recommended dietary allowance (RDA) and tolerable upper levels (UL) [46]. Most major elements such as Mg, Ca, K and Na, and heavy metals such as Mn, Zn, Fe, Cu and Cr as determined by Shelembe [46] are within the permissible limit defined by FAO/WHO.

Pegel and Rogers [69] isolated delphinidin, ellagic acid, epifriedelinol, friedeline, gallic acid, taraxerole and taraxerone from stem bark and caffeic acid from the leaves of *B. micrantha* (Table 3). Twenty four essential oils were isolated from *B. micrantha* stem bark by Green et al. [78] (Table 3). Akinyeye and Olatunya [72] isolated phytic acid from the bark of *B. micrantha* (Table 3). Shelembe [46] isolated cycloartenol, cycloartenol acetate, ergosterol, stigmast-8(14)-en-3-ol, and 5β,6β-epoxy-7-bromocholestan-3-one from fruits and acacic acid lactone, quercetin, quercetin-3-*O*-glucoside, oleanolic acid from the stem bark and leaves of *B. micrantha* (Table 3). Similarly, Shelembe et al. [77] isolated quercetin, quercetin-3-*O*-glucoside, oleanolic acid and acacic acid lactone from the stem bark and leaves while cycloartenol, cycloartenol acetate, ergosterol and stigmast-8(14)-en-3-ol were isolated from fruits of *B. micrantha* (Table 3).

Munayi [26] isolated *trans*-triacontyl-4-hydroxy-3-methoxyxinnamte, betulinic acid, catechin and friedelin from the stem bark of *B. micrantha* (Table 3). Some of these compounds, particularly alkaloids, essential oils, flavonoids, phenolics and tannins could be responsible for some of the ethnomedicinal uses of *B. micrantha* listed in Table 1.

## 6. Pharmacological Properties of *B. micrantha*

The following activities have been reported from *B. micrantha*: anthelmintic [79], antibacterial [19,32,36,74,80,81,82,83], anticonvulsant and sedative [84], antidiabetic [85], antidiarrhoeal [25], antifungial [36,83], anti-*Helicobacter pylori* [70,81,82], antimycobacterial [78,86], antinociceptive [87], antioxidant [77,85,87,88,89], antiplasmodial [59,90,91], antischistosomal [92], antiviral [39,40], hepatoprotective [88], insecticidal [93], β-lactamase inhibitory [94], toxicity and cytotoxicity [87,95,96,97,98,99].

### 6.1. Anthelmintic

Waterman et al. [79] evaluated the anthelmintic activities of aqueous and organic bark extract of *B. micrantha* using a standard motility assay against a levamisole resistant strain of the nematode *Caenorhabditis elegans*. The degree of activity of extracts was presented as average percentage of worm death and statistically compared to a negative control. *B. micrantha* aqueous and organic bark extracts showed 89.4% and 80.7% dead worms higher than the negative control [79]. These anthelmintic evaluations are of importance in the traditional use of *B. micrantha* against tape worms in Cameroon [32], Ghana [34], Kenya [38,60], Nigeria [41], South Africa [2,25], Tanzania [9] and Uganda [15] and will also play an important role in future research focusing on control and management of worm infections in sub-Saharan Africa.

### 6.2. Antibacterial

Samie et al. [80] evaluated the antibacterial activities of *B. micrantha* methanol, acetone and hexane bark, roots and seeds extracts against *Aeromonas hydrophila*, *Bacillus cereus*, *Bacillus pumilus*, *Bacillus subtilis*, *Enterobacter cloacae*, *Enterococcus fecalis*, *Escherichia coli*, *Klebsiella pneumoniae*, *Pantoea agglomerans*, *Proteus mirabilis*, *Pseudomonas aeruginosa*, *Salmonella cholerae-suis*, *Serratia marcescens*, *Staphylococcus aureus* and *Shigella flexneri* using the disc diffusion and the micro-dilution methods. The extracts of the bark, roots and seeds of *B. micrantha* gave diameter of zone of inhibition ranging from 8–14 mm against all pathogenic organisms except *K. pneumoniae* and *S. cholerae-suis* [80]. The minimal inhibitory concentration (MIC) values of methanol, acetone and hexane bark, roots and seeds extracts against the tested bacteria were within the range of 1.5 mg/mL to >12 mg/mL [80]. Similarly, Steenkamp et al. [19] evaluated antibacterial activities of methanol bark extract of *B. micrantha* against *E. coli*, *P. aeruginosa*, *S. aureus* and *Staphylococcus epidermidis* using the plate-hole diffusion and broth micro-dilution methods. None of the extracts showed activity against *E. coli* and *P. aeruginosa* while MIC values between 1.25 mg/mL and 5.00 mg/mL were obtained against *S. aureus* and *S. epidermidis* [19]. Gangoué-Piéboji et al. [32] evaluated the antibacterial activities of *B. micrantha* methanol stem bark extracts against clinically proved beta-lactam-resistant bacteria *Acinetobacter baumannii*, *Enterobacter aerogenes*, *E. cloacae*, *Enterococcus hirae*, *Enterococcus spp.*, *E. coli*, *Klebsiella oxytoca*, *K. pneumoniae*, *P. aeruginosa*, *S. marcescens* and *S. aureus* by using disc-diffusion and agar-dilution assays. The methanol extracts demonstrated broad spectrum activity against all bacteria tested with inhibition zones in the range of 14–27 mm. The MIC values of methanol extracts against the tested bacteria were found to range from 1.25 mg/mL to 10 mg/mL [32]. Adefuye et al. [81] evaluated antibacterial activities of *B. micrantha* dichloromethane, ethyl acetate, acetone, ethanol, methanol and methanol hydroxide stem bark extracts against *Salmonella typhimurium*, *Shigella sonnei* and *S. aureus* using agar-well diffusion method with ciprofloxacin as control. Zone diameters of inhibition ranged from 0 to 28 mm for the six extracts and 29 to 38 mm for ciprofloxacin. The MIC_50_ values ranged from 0.078 mg/mL to 1.25 mg/mL [81]. Mabeku et al. [36] evaluated the antibacterial activities of methanol, ethanol, hexane, ethyl acetate, aqueous, mixture of methanol and water, and mixture of ethanol and water stem bark extracts of *B. micrantha* against *B. cereus*, *Citrobacter freundii*, *E. cloacae*, *E. coli*, *K. pneumoniae*, *Morganella morganii*, *P. agglomerans*, *Proteus vulgaris*, *P. mirabilis*, *P. aeruginosa*, *Salmonella typhi*, *Shigella dysenteriae*, *S. flexneri*, *S. aureus* and *Streptococcus feacalis* using the disc diffusion assay and broth micro-dilution methods. The best antibacterial activity was demonstrated against *S. typhi* with MIC value of 50 μg/mL and minimal bactericidal concentration (MBC) value of 400 μg/mL [36]. In a different study, Adefuye and Ndip [82] evaluated antibacterial activities of ethyl acetate extract of stem bark of *B. micrantha* against *S. typhimurium*, *S. sonnei* and *S. aureus* with ciprofloxacin as a positive control. The MIC_50_ values ranged from 0.0048 mg/mL to 0.312 mg/mL [82]. Traore et al. [83] evaluated antibacterial activities of methanolic stem bark extracts of *B. micrantha* against *E. coli*, *Mycobacterium chelonae* and *S. aureus*. The methanolic extract of *B. micrantha* demonstrated weak activity against *E. coli* with IC_50_ values of 64 μg/mL [83]. Douglas and Gitonga [74] evaluated antibacterial activities of methanol and ethyl acetate leaf extracts of *B. micrantha* against *E. coli*, *S. typhi* and *S. aureus* using micro-broth dilution assay. Methanol extract of *B. micrantha* exhibited a zone of inhibition of 7 to 19 mm against *S. aureus* and *S. typhi* respectively [74]. These documented antibacterial properties of *B. micrantha* corroborate the traditional uses of the species against bacterial infections such as cough in Cameroon, DRC, Nigeria and Zimbabwe [16,18,21,22,28,32,33], diarrhoea in Cameroon, DRC, Kenya, Nigeria, South Africa and Tanzania [2,9,16,22,25,32,36,38,39,40,41], dysentery in Burundi, Cameroon, DRC, Gambia and Nigeria [16,33,36,42,43], sexually transmitted diseases in Guinea-Bissau and Kenya [47,60,63], stomach ache in Guinea-Bissau, Kenya, Nigeria, South Africa and Tanzania [38,41,47,48,51,60], syphilis in Sierra Leone and Uganda [61,65] and venereal diseases in Kenya and South Africa [2,23,25,38].

### 6.3. Anticonvulsant and Sedative

Bum et al. [84] evaluated the anticonvulsant effects of crude stem bark extracts of *B. micrantha* using mice model maximal electroshock (MES), strychnine (STR), pentylenetetrazol (PTZ), picrotoxin (PIC), isonicotinic hydrazide acid (INH)-induced convulsions and diazepam-induced sleep in assessing the sedative effects. Results showed that *B. micrantha* stem bark extracts at the doses of 34 and 67 mg/kg protected 100%, 80%, 80% and 80% of mice from PIC, STR, PTZ and MES-induced seizures, respectively [84]. *B. micrantha* also delayed the onset to seizures in the INH test. The decoctions of *B. micrantha* possess sedative and anticonvulsant activities and these results corroborate the use of the species as herbal medicine for epilepsy and insomnia in Cameroon [55].

### 6.4. Antidiabetic

Adika et al. [85] evaluated the antidiabetic activities of methanol leaf extract of *B. micrantha* using alloxan-induced diabetic mice in vivo and in vitro. The methanol leaf extract at the dose of 250, 500, and 1500 mg/kg showed remarkable time-dependent decrease in blood glucose level in alloxan-induced diabetic mice. Adika et al. [85] found that there was no significant difference between the extract-treated groups and the groups treated with 10 mg/kg of distilled water and glibenclamide (2 mg/kg) respectively. Adika et al. [85] found that after six hours posttreatment, the blood glucose level for the groups treated with 250 mg/kg of *B. micrantha* and glibenclamide (2 mg/kg) were lower than the normal blood glucose level for the groups before the induction of diabetes. Even in its crude form, the effects were comparable to that of glibenclamide, an oral sulfonylurea with proven antidiabetic activity. These findings demonstrate that *B. micrantha* has antidiabetic effects on experimental model of diabetes in mice and validate its use in Cameroon, Guinea and Nigeria as a traditional medicine for the treatment of diabetes mellitus [34,35,36,37]. This finding suggests that the leaf extract could be a potential source of a novel antidiabetic for the management of diabetes mellitus.

### 6.5. Antidiarrhoeal

Lin et al. [25] evaluated anti-diarrhoeal activities of aqueous and methanolic bark extract of *B. micrantha* against different experimental models of diarrhoea in rats as well as bacteria that cause diarrhoea such as *E. coli*, *Plesiomonas shigelloides*, *Salmonella virchow* and *S. dysenteriae* and *S. flexneri*. The methanolic bark extract of *B. micrantha* demonstrated weak inhibitory activities against *P. shigelloides* and *S. flexneri* [25]. Based on the results in experimental rat models, there were significant reductions in faecal output and frequency of droppings when plant extracts were administered compared with castor-oil treated rats [25]. All plant extracts also significantly retarded the propulsion of charcoal meal and significantly inhibited the PGE(2)-induced enteropooling [25]. These findings by Lin et al. [25] corroborate the species’ antidiarrhoeal potential and its traditional use in the treatment of diarrhoea, dysentery and other gastro-intestinal problems in Burundi [43], Cameroon [21,32,46], DRC [16,22], Gambia [42], Guinea-Bissau [47,48], Kenya [38,60], Nigeria [33,41], South Africa [2,25,39,40] and Tanzania [9,51].

### 6.6. Antifungal

Mabeku et al. [36] evaluated the antifungal activities of methanol, ethanol, hexane, ethyl acetate, aqueous, mixture of methanol and water, and mixture of ethanol and water stem bark extracts of *B. micrantha* against *Candida albicans* and *Candida glabrata* using the disc diffusion assay and broth micro-dilution methods. The ethanol extract of stem bark of *B. micrantha* gave a diameter zone of inhibition of 10 mm against *C. glabrata* [36]. Similarly, Traore et al. [83] evaluated antifungal activities of methanolic stem bark extracts of *B. micrantha* against *Aspergillus fumigatus*, *C. albicans* and *Trichophyton rubrum*. Traore et al. [83] used the following arbitrary scale in assessing the level of antimicrobial activity: strong (IC_50_ ≤ 10 μg/mL), good (10 μg/mL < IC_50_ ≤ 20 μg/mL), moderate (20 μg/mL < IC_50_ ≤ 40 μg/mL), weak (40 μg/mL < IC_50_ ≤ 64 μg/mL) and inactive (IC_50_ ≥ 64 μg/mL). The methanolic extract of *B. micrantha* demonstrated weak activity against *A. fumigatus* with IC_50_ value of 64 μg/mL [83]. These documented antifungal properties correlate with traditional applications of *B. micrantha* against various skin infections in Cameroon [29], Kenya [62] and Tanzania [51].

### 6.7. Anti-Helicobacter pylori

Adefuye et al. [81] evaluated anti-*Helicobacter pylori* activities of *B. micrantha* dichloromethane, ethyl acetate, acetone, ethanol, methanol and 40% methanol hydroxide stem bark extracts against *Helicobacter pylori* using agar-well diffusion method with ciprofloxacin as control. Zone diameters of inhibition ranged from 0 to 18 mm for the extract and 29 to 38 mm for ciprofloxacin. The MIC_50_ values ranged from 0.312 mg/mL to 0.625 mg/mL [81]. Okeleye et al. [70] evaluated anti-*Helicobacter pylori* activities of ethyl acetate, acetone, aqueous and methanol extracts of stem bark of *B. micrantha* against *Helicobacter pylori* using clarithromycin, metronidazole and amoxicillin as controls. The inhibition zone diameters ranged from 0–23 mm for all the extracts in comparison to 0–35 mm observed for clarithromycin [70]. Marked susceptibility of *H. pylori* strains (100%) was observed for the acetone extract, followed by ethyl acetate extract at 93.5% and clarithromycin had susceptibility of 58.1% [70]. The MIC_50_ values ranged from 0.0048 to 0.313 mg/mL while the MIC_90_ values ranged from 0.0048 to 2.5 mg/mL for the acetone and ethyl acetate extracts [70]. Adefuye and Ndip [82] evaluated anti-*Helicobacter pylori* activities of ethyl acetate extract of stem bark of *B. micrantha* against *H. pylori* with ciprofloxacin as positive control. *B. micrantha* extracts showed some activity with MIC_50_ values ranging from 1.25 mg/mL to 5.0 mg/mL [82]. The ethyl acetate extract could thus be a potential source of lead molecules for the design of new anti-*Helicobacter pylori* therapies. These findings are significant since bark, leaf and root decoctions of *B. micrantha* are widely used as traditional remedies for gastric ulcers in Cameroon [32], DRC [16] and South Africa [46].

### 6.8. Antimycobacterial

Green et al. [86] evaluated the antimycobacterial activities of acetone, methanol, hexane and ethanol leaf extracts of *B. micrantha* against *Mycobacterium tuberculosis* using a tetrazolium microplate assay to determine the minimum inhibitory concentration (MIC). Acetone extract was active against *M. tuberculosis* with a MIC value of 25 µg/mL [86]. In another study, Green et al. [78] evaluated the antimycobacterial activities of the *n*-hexane sub-fraction of ethyl acetate fractions from acetone extracts of *B. micrantha* stem barks using the resazurin microplate assay against *M. tuberculosis*. The *n*-hexane fraction showed 20% inhibition of *M. tuberculosis* H37Ra and almost 35% inhibition of *M. tuberculosis* isolate resistant to all first-line drugs at 10 μg/mL [78]. The primary ethyl acetate fraction showed MIC value of 8.25 μg/mL against H37Ra *M. tuberculosis* strain [78]. The fraction also inhibited the growth of *M. tuberculosis* isolate resistant to isoniazid (INH), ethambutol (EMB), streptomycin (STM) and rifampicin (RIF) at a concentration of 50 μg/mL [78]. These preliminary evaluations done by Green et al. [78,86] serve as scientific validation for the use of *B. micrantha* in traditional medicine for treatment of respiratory systems such as chest complaints and cough in Cameroon [21,28,32], DRC [16,22], Nigeria [33] and Zimbabwe [18].

### 6.9. Antinociceptive

Onoja et al. [87] evaluated the antinociceptive effects of the hydromethanolic extract of *B. micrantha* stem bark. The antinociceptive effects of *B. micrantha* extracts at the doses of 50, 100 and 200 mg/kg were investigated using male Wistar rats via acetic acid-induced writhing reflex and tail flick methods and the effects of *B. micrantha* on thiopentone-induced narcosis was also investigated. The *B. micrantha* extracts produced a significant dose-dependent decrease in the mean number of abdominal constriction in the acetic acid-induced writhing reflex when compared to the negative control. Both the extract (200 mg/kg) and paracetamol (400 mg/kg) produced 61.85% and 73.08% inhibition of writhing reflex, respectively [87]. *B. micrantha* and pentazocine (3 mg/kg) caused a significant increase in the pain reaction time (PRT) in treated rats when compared to the negative control group in tail flick method. The pretreatment of the rats with *B. micrantha* at different doses increased the normal sleeping time of thiopentone from 69.33 ± 7.31 min to an average of 105.33 ± 11.88 min [87]. The results of the effect of the extract on acetic acid-induced writhing reflex suggest that the extract may have a peripheral pain relieving effect mediated through the inhibition of cyclooxygenase and prostaglandin synthesis. These findings support the traditional use of *B. micrantha* in the management of pain, for example as remedy for abdomninal pain in DRC and Uganda [15,16], epigastric pain and sore eyes in Tanzania [45], muscle pain in Cameroon [55], painful eyes, joints and as pain killer in South Africa [2,23,25,39,46,54] and wounds in DRC, Sierra Leone and South Africa [2,16,23,24,25,65].

### 6.10. Antioxidant

Nwaehujor and Udeh [88] evaluated the antioxidant activities of the methanolic leaf extracts of *B. micrantha* using the DPPH (2,2-diphenyl-1-picrylhydrazyl) and ferric reducing antioxidant power (FRAP) assay methods. The DPPH assay showed 98% antioxidant activity at concentration of 400 µg/mL while FRAP values were 0.016, 0.39, 0.455, 0.601 and 1.382 µM at 10, 50, 100, 200 and 400 µg/mL respectively [88]. Adika et al. [85] evaluated antioxidant activities of methanol leaf extracts of *B. micrantha* using DPPH spectrophotometric assay at the concentration of 400 μg/mL. The ferric reducing antioxidant power showed a significant concentration-dependent increase in the total antioxidant power. Nwaehujor et al. [89] evaluated antioxidant activities of the methanolic leaf extracts of *B. micrantha* using the DPPH and FRAP assay methods with ascorbic acid as reference standard. The methanol extracts of *B. micrantha* leaves gave 98% antioxidant activity at a maximal test concentration of 400 µg/mL while ascorbic acid produced a comparatively reduced percentage value of 79% at the same concentration [89]. In the FRAP assay, *B. micrantha* leaf extracts produced 1.39 µM at 400 µg/mL but 1000 µg/mL of ascorbic acid had a standard FRAP value of 2.0 µM [89]. Onoja et al. [87] evaluated the antioxidant activities of the hydromethanolic extract of *B. micrantha* stem bark using 2,2-diphenyl-1-picrylhydrazyl (DPPH) photometric assay. *B. micrantha* extract produced concentration-dependent increase in percentage antioxidant activity in DPPH photometric assay. The *B. micrantha* extract demonstrated a potent antioxidant activities with 50% inhibitory concentration (IC_50_) of <25 μg/mL concentration in DPPH photometric assay [87]. These results suggest that *B. micrantha* has antioxidant potential. Shelembe et al. [77] evaluated the antioxidant activities of fruit methanol extracts of *B. micrantha* using the DPPH stable free radical method using ascorbic acid as control. The IC_50_ value for the methanol extract of the stem bark of *B. micrantha* was 150 μg/mL while that of the standard ascorbic acid was 41 μg/mL indicating the high antioxidant potential of the plant, especially the stem bark [77]. The scavenging effect of fruit extract increased with increasing concentrations, with the extract exhibiting appreciable scavenging activity at 1000 μg mL^−^^1^ with the scavenging ability 85.5% [77]. These antioxidant activities are probably due the presence of flavonoids and phenolic compounds [100].

### 6.11. Antiplasmodial

Clarkson et al. [90] evaluated antiplasmodial activities of *B. micrantha* aqueous, dichloromethane, dichloromethane and methanol (1:1) twig extracts against *Plasmodium falciparum* using the parasite lactate dehydrogenase (pLDH) assay. *B. micrantha* dichloromethane and methanol (1:1) extract showed a weak activity with IC_50_ value of 59.3 µg/mL [90]. Similarly, Ajaiyeoba et al. [91] evaluated antiplasmodial activities of *B. micrantha* methanol leaf extracts against *P. falciparum* using the lactate dehydrogenase (pLDH) assay. *B. micrantha* extract showed a weak activity with IC_50_ value of 158.7 µg/mL [91]. Nondo et al. [59] evaluated antiplasmodial activities of *B. micrantha* stem bark ethanol extracts against chloroquine-resitant *P. falciparum* strain using the parasite lactase dehydrogenase method. *B. micrantha* extracts inhibited malaria parasite by 71.87% growth inhibition rate at 100 µg/mL on *P. falciparum* strain. Although *B. micrantha* extracts are characterized by weak antiplasmodial activities [59,90,91], these results provide compelling evidence for the rationale of the species as malaria remedy in Guinea [57], Ivory Coast [58], Kenya [56] and Tanzania [20,59].

### 6.12. Antischistosomal

Waiganjo et al. [92] evaluated the antischistosomal activities of hexane, methanol and water extract of *B. micrantha* bark on Swiss white mice infected with *Schistosoma mansoni* with praziquantel as control. There were no significant differences between praziquantel which showed worm reduction percentages of 75.2% against 48.7% and 63.4% demonstrated by hexane and water extracts respectively [92]. These findings demonstrated that *B. micrantha* extracts were able to protect the mice from schistosomiasis infection.

### 6.13. Antiviral

Bessong et al. [39] evaluated *B. micrantha* leaf methanol extracts against human immunodeficiency type 1 reverse transcriptase by assessing inhibition of the RNA dependent DNA polymerase activity by measuring the degree of incorporation of methyl-3H thymidine triphosphate using polyadenylic acid. Ribonuclease H activity was evaluated by measuring the extent of degradation of a radiolabelled RNA in an RNA/DNA hybrid by reverse transcriptase. The methanol extract of the leaves of *B. micrantha* inhibited the polymerase with IC_50_ value of 23.5 μg/mL and the ribonuclease H with IC_50_ value of 18.9 μg/mL [39]. In another study, Bessong et al. [40] evaluated antiviral activities of *B. micrantha* root methanol extracts for activity against HIV-1 reverse transcriptase (RT) and integrase (IN). The *n*-butanol fraction obtained from the crude methanol extracts of *B. micrantha* inhibited the RNA-dependent-DNA polymerization (RDDP) activity of HIV-1 RT with an IC_50_ of 7.3 µg/mL. More pharmacological evaluations are required as *B. micrantha* is widely used against viral infections such as HIV/AIDs in Kenya [52], yellow fever in Tanzania [20] and other infectious diseases such as diarrhoea, dysentery, sexually transmitted infections (STIs) and skin infections.

### 6.14. Hepatoprotective

Nwaehujor and Udeh [88] evaluated hepatoprotective activities of the methanolic leaf extracts of *B. micrantha* on paracetamol induced liver damage in Wistar rats through measuring alanine aminotransaminase (ALT), aspartate aminotransferase (AST), alkaline phosphate (ALP), bilirubin and total protein. *B. micrantha* extract significantly decreased the level of AST in the rats given PCM, reduced ALP and total bilirubin while total protein was significantly increased [88]. Nwaehujor and Udeh [88] also found that the ethyl acetate extract of *B. micrantha* had better hepatoprotective effects on the Wistar rats than the known silymarin especially at 300 mg/kg. Therefore, *B. micrantha* has the ability to help in the regeneration of damaged hepatic cells and can be useful in the treatment and management of hepatic diseases.

### 6.15. Insecticidal

Adesina et al. [93] evaluated the insecticidal activities of aqueous leaf extracts of *B. micrantha* against *Podagrica uniforma* (Jacoby) and *Nisotra dilecta* (Jacoby) insect pests of okra (*Abelmoschus esculentus* (L.) Moench) with synthetic insecticide cypermethrin as control. Yield parameters such as number of fruits and fruit weight were collected on insect population before treatment application and three days after spraying of insecticides at 28, 35, 42, 56 days after planting (DAP). There were no significant differences between mean number of *P. uniforma* and *N. dilecta* counted on okra plants after 56 DAP which were 0.80 ± 0.10 and 0.47 ± 0.17 for *B. micrantha* and cypermethrin respectively. There were no significant differences in mean yield of okra fruits after application of *B. micrantha* and cypermethrin which stood at 0.93 ± 0.3 and 2.20 ± 0.23 respectively [93]. Results obtained showed that *B. micrantha* extracts exhibited effectiveness in reducing the insect population and improved *A. esculentus* fruit yield compared to cypermethrin. Therefore, *B. micrantha* crude extracts could be explored as promising insecticidal agents to provide valuable alternatives to chemical control of insect infestation on *A. esculentus* [93]. These findings indicate that *B. micrantha* has insecticidal properties corroborating traditional use of the bark and leaf decoction as insecticide in Tanzania [51].

### 6.16. β-Lactamase Inhibitory

Gangoué-Piéboji et al. [94] evaluated the anti-β-lactamase activities of methanolic stem bark extracts of *B. micrantha* by assessing the inhibition activities (over 90%) against four classes of β-lactamases, namely TEM-1, OXA-10, IMP-1 and P99. *B. micrantha* extracts had strong inhibition activities of 99.2% and 92.0% against OXA-10 and P99 respectively. After elimination of tannins, the extracts of *B. micrantha* were tested further for anti-β-lactamase activities with OXA-10 demonstrating potent inhibitory activity with 50% inhibitory concentration (IC_50_) value of 0.02 mg/mL [94]. Further research aimed at isolating and elucidating the chemical structure of the active constituents of *B. micrantha* will provide useful leads in the search and development of β-lactamase inhibitors.

### 6.17. Toxicity and Cytotoxicity

Ajaiyeoba et al. [95] reported the cytotoxicity effects of 20 plants used in Nigeria as antimalarials. The 50% lethal concentration (LC_50_ value) at 95% confidence interval was calculated with the brine shrimp lethality assay for each plant methanol extract. *B. micrantha* leaf extract showed the least LC_50_ value of >9.0 × 10^6^ µg/mL [95]. Moshi et al. [97] evaluated toxicity of dichloromethane and/or ethanol root extracts of *B. micrantha* using brine shrimp toxicity with dimethyl sulphoxide (DMSO) and seawater as negative controls. *B. micrantha* ethanol root extract was found to be mildly toxic with concentration killing fifty percent of the larvae (LC_50_) of 30 μg/mL [97]. Onoja et al. [87] assessed oral acute toxicity of hydromethanolic stem bark extract of *B. micrantha* using the OECD guideline, the up-and-down-procedure by orally dosing five male Wistar rats with 2000 mg/kg of *B. micrantha* and observing the rats for 48 h for signs of toxicity and death. Oral administration of the *B. micrantha* extract was well tolerated at the dose of 2000 mg/kg and no death and clinical signs of toxicity were observed [87]. Similarly, Osebe et al. [99] evaluated toxicity of water stem bark extract of *B. micrantha* using brine shrimp lethality test for the determination of LC_50_ and the water extract with LC_50_ of 77 μg/mL was deemed toxic.

Steenkamp et al. [96] evaluated the cytotoxicity of *B. micrantha* using human adenocarcinoma cells of the cervix (HeLa), human breast cells (MCF-12A), lymphocytes (both resting and stimulated) and primary porcine hepatocytes. Steenkamp et al. [96] also determined acute systemic toxicity of *B. micrantha* extracts using the luminescent bacteria *Vibrio fischerii* and the vertebrate *Poecilia reticulata* (guppy). The 50% inhibition of proliferation (IC_50_) of *B. micrantha* in HeLa cells was 8.9 μg/mL, IC_50_ was 24.2 μg/mL in MCF-12A cells and the positive control, cisplatin had IC_50_ value of 1.14 μg/mL and 0.21 μg/mL for the HeLa and MCF-12A cells respectively. Toxicity was found to be concentration dependent when HeLa and MCF-12A cells were exposed to *B. micrantha* extracts. *B. micrantha* extracts resulted in 100% mortality of the guppies [96]. Similarly, Omosa et al. [98] evaluated the cytotoxicity of dichloromethane and methanol (1:1) extract of *B. micrantha* leaves and stem bark using the resazurin reduction assay against CCRF-CEM leukemia cell line. The dichloromethane and methanol extract of *B. micrantha* leaves and stem bark displayed cytotoxicity towards leukemia CCRF-CEM cells with IC_50_ value of 9.43 µg/mL and 23.5 µg/mL respectively [98]. These studies show that *B. micrantha* extracts are cytotoxic and possess acute systemic toxicity. According to Verdcourt and Trump [101], *B. micrantha* is poisonous as death of a patient has been reported, four hours after the ingestion of a cough mixture made from *B. micrantha* herbal concoction in Kenya.

## 7. Conclusions

The historical traditional usage of *B. micrantha* as herbal medicine throughout its distribution range, the documented phytochemistry and pharmacological properties call for detailed pharmacokinetics and clinical research on the species and its pharmaceutical and food products. At the moment there is insufficient evidence to interpret the documented ethnomedicinal uses with specific chemical mechanisms associated with some of the documented pharmacological properties. Future research should identify the bioactive compounds, details of their molecular modes or mechanisms of action, pharmacokinetics and physiological pathways for specific bioactives of *B. micrantha. B. micrantha* has been categorized as poisonous by Verdcourt and Trump [101], toxicity and cytotoxicity studies conducted by Steenkamp [96], Moshi et al. [97], Omosa et al. [98] and Osebe et al. [99] appear to suggest that the species is toxic and may cause damage to genetic material and therefore, has potential to cause long-term damage in patients when administered as herbal medicines. There is need for rigorous toxicological and clinical studies aimed at identification of poisonous compounds, associated pharmacological activities and the side effects that are likely to be caused when *B. micrantha* is used as herbal medicine. Therefore, future research should focus on dosage range that is safe for humans and evaluation of target-organ toxicity.

## Figures and Tables

**Table 1:** Ethnomedicinal uses of *B. micrantha* in tropical Africa.

Use	Plant Parts Used	Country Practiced	References
Abdominal pain	Bark, leaf and root decoction taken orally	DRC, Uganda	[15,16]
Abortifacient	Bark decoction taken orally	South Africa, Zimbabwe	[5,17,18,19]
Amenorrea	Root decoction taken orally	Tanzania	[20]
Amoebic dysentery	Bark decoction taken orally	Cameroon	[21]
Anaemia	Bark, leaf or root decoction taken orally	DRC	[16,22]
Anaesthetic	Root decoction taken orally mixed with roots of *Vangueria infausta* Burch. (Rubiaceae) and *Dichrostachys cinerea* (L.) Wight & Arn. (Fabaceae)	South Africa	[2]
Burns	Bark decoction applied on affected body part	South Africa	[2,23,24,25]
Cancer	Bark decoction taken orally	Kenya	[26]
Cervical, breast, skin colorectal cancer	Leaves, roots and stem bark taken orally	Kenya	[27]
Chest complaints	Bark decoction taken orally mixed with *Pittosporum viridiflorum* Sims (Pittosporaceae)	Cameroon	[28]
Conjunctivitis	Bark, leaf or root decoction applied to eyes	Cameroon, DRC	[16,29]
Constipation	Bark, leaf or root decoction taken orally	DRC, Gambia, Ivory Coast	[16,30,31]
Cough	Bark, leaf or root decoction taken orally	Cameroon, DRC, Zimbabwe	[16,18,21,22,32]
Cough	Bark decoction taken orally mixed with *P. viridiflorum*	Cameroon	[28]
Cough	Bark mixed with *Capsicum frutescens* L. (Solanaceae)	Nigeria	[33]
Dermatitis	Bark, leaf or root decoction applied to affected body part	Cameroon	[29]
Diabetes mellitus	Bark decoction taken orally	Cameroon, Guinea, Nigeria	[34,35,36,37]
Diarrhoea	Bark, leaf or root decoction taken orally	Cameroon, DRC, Kenya, Nigeria, South Africa, Tanzania	[2,9,16,22,25,32,36,38,39,40,41]
Dysentery	Bark, leaf or root decoction taken orally	Burundi, Cameroon, DRC, Gambia	[16,36,42,43]
Dysentery	Bark taken orally mixed with *Antrocaryon klaineanum* Pierre (Anacardiaceae) and *Treculia africana* Decne. ex Trécul (Moraceae)	Nigeria	[33]
Dysmenorrhea	Root decoction taken orally	Tanzania	[20]
Emetic	Bark infusion taken orally	South Africa	[44]
Epigastric pain	Root decoction taken orally	Tanzania	[45]
Eye diseases	Bark decoction applied to the eyes	Cameroon	[32]
Fever	Bark, leaf or root decoction taken orally	DRC, South Africa	[16,23]
Gastric ulcers	Bark, leaf or root decoction taken orally	Cameroon, DRC, South Africa	[16,32,46]
Gastro-Intestinal ailments	Bark decoction taken orally	South Africa	[25]
Guinea worm	Leaf decoction taken orally	Ghana	[34]
Haemorrhoids	Bark, leaf or root decoction taken orally	DRC, Guinea-Bissau	[16,22,47,48]
Headache	Bark, leaf or root decoction sometimes with oil or butter rubbed into scalp	DRC, Nigeria, South Africa, Tanzania	[9,16,23,45,49,50]
Hernia	Leaf or bark decoction taken orally	Tanzania	[51]
HIV/AIDs	Bark decoction taken orally	Kenya	[52]
Induce labour pains	Bark decoction taken orally	Nigeria	[53]
Infertility	Bark decoction taken orally	Cameroon	[36]
Infertility	Root decoction taken orally mixed with roots of *P. africanum* Sond. and *Ochna* spp. (Ochnaceae)	South Africa	[54]
Insomnia	Bark decoction taken orally	Cameroon	[55]
Malaria	Bark, leaf or root decoction taken orally	Guinea, Ivory Coast, Kenya, South Africa, Tanzania	[20,23,56,57,58,59]
Migraine	Bark, leaves or root decoction taken orally	Nigeria	[49,50]
Muscle pain	Bark decoction rubbed on painful muscles	Cameroon	[55]
Numbness of feet	Bark decoction rubbed on feet	Cameroon	[55]
Painful joints	Bark decoction rubbed on painful joints	Kenya, South Africa	[25,60]
Pain killer	Roots taken orally mixed with roots of *V. infausta* and *D. cinerea*	South Africa	[54]
Paralysis	Bark decoction taken orally	South Africa	[25]
Physical weakness	Bark decoction taken orally	Cameroon	[55]
Pre-Hepatic jaundice	Bark decoction taken orally	Uganda	[61]
Purgative for poisoning	Leaf decoction taken orally	Ivory Coast	[31]
Rheumatism	Bark, leaf or root decoction taken orally	Cameroon, Tanzania	[29,51]
Sexual weakness	Bark decoction taken orally	Cameroon	[55]
Skin eruption	Bark or leaf decoction applied to affected body part	Kenya, Tanzania	[51,62]
Spleen enlargement	Bark or leaf decoction taken orally	Tanzania	[51]
Sexually transmitted diseases (STDs)	Bark, leaf or root decoction taken orally	Guinea-Bissau, Kenya	[47,60,63]
Scorpion bite	Bark decoction applied to affected body part	Ethiopia	[64]
Sore eyes	Bark, leaf sap or leaf decoction applied on eyes	South Africa, Tanzania	[2,23,39,45,46]
Stomach ache	Bark, leaf or root decoction taken orally	Guinea-Bissau, Kenya, Nigeria, South Africa, Tanzania	[38,39,40,41,47,48,51,60]
Syphilis	Bark decoction taken orally mixed with *Mangifera indica* L. (Anacardiaceae)	Sierra Leone	[65]
Syphilis	Bark or leaf decoction taken orally	Uganda	[61]
Tape worms	Bark or leaf decoction taken orally	Cameroon, Kenya, Nigeria, South Africa, Tanzania, Uganda	[2,9,15,25,32,38,41,60]
Toothache	Bark decoction applied to affected teeth	DRC, South Africa	[2,22,23,25]
Tumor	Stem bark decoction taken orally	Kenya	[26]
Venereal diseases	Bark or leaf decoction taken orally	Kenya, South Africa	[2,23,25,38]
Wounds	Bark, leaf or root decoction applied to affected body part	DRC, Sierra Leone, South Africa	[2,16,23,24,25,65]
Yellow fever	Root decoction taken orally	Tanzania	[20]
Ethnoveterinary medicine		Burkina Faso, DRC, Ethiopia, Kenya, Uganda	[14,60,66,67,68]

**Table 2:** Nutritional composition of *B. micrantha* bark, fruits and leaves.

Nutritional Composition	Values	Dietary Reference Intake (DRI) (mg/day)		References
		Recommended Dietary Allowance (RDA)	Tolerable Upper Intake Level (UL)	
Ash	3.2 ± 0.46 g/100 g	-	-	[46]
Carbohydrate	1.7 g/100 g	-	-	[46]
Ca	543 µg g^−1^	1000–1300	2500	[46]
Co	6.8 µg g^−1^	-	-	[46]
Cr	3 µg g^−1^	0.024–0.035	1000	[46]
Cu	9 µg g^−1^	0.9	8.0	[46]
Cyanogenic glycosides	810 mg/100 g	-	-	[73]
Fe	166 µg g^−1^	8–15	45.0	[46]
K	87.94 mg/100 g	3000	3000	[72]
Mg	859 µg g^−1^	310–320	350	[46]
Mn	414 µg g^−1^	1.6–3.0	9.0	[46]
Moisture content	90.1 ± 0.60 g/100 g	-	-	[46]
Na	254.8 mg/100 g	2300	2300	[72]
Ni	3 µg g^−1^	-	-	[46]
Oil	0.9 ± 0.01 g/100 g	-	-	[46]
Oxalate	5.84 g/100 g	-	-	[73]
P	2545.4 mg/100 g	-	-	[72]
Pb	1 µg g^−1^	-	-	[46]
Phytic acid	0.5%	-	-	[72]
Protein	4.1 ± 0.13 g/100 g	-	-	[46]
Saponin	10.6%	-	-	[73]
Tannin	1160 mg/100 g	-	-	[73]
Zn	226 µg g^−1^	8–11	34.0	[46]

**Table 3:** Phytochemical compounds isolated from the bark, leaves and fruits of *B. micrantha*.

Phytochemical Compounds	Plant Parts	Method of Compound Characterization	References
**Alkaloid**			
Stigmast-8(14)-en-3-ol	Fruits	Gas chromatography-mass spectrometry (GC-MC)	[46,77]
**Anthocyanidin**			
Delphinidin	Bark	Thin-Layer chromatography (TLC)	[69]
**Essential oil**			
*N*(β)-Benzyl-14-(carboxymethyl)	Bark	GC-MS	[78]
Benzene, 1.3-bis (3-phenoxyphenoxy)	Bark	GC-MS	[78]
2-Phenyl-2-tipyl-acenapthenone	Bark	GC-MS	[78]
α-Pinene	Bark	GC-MS	[78]
Camphene	Bark	GC-MS	[78]
1,8-Cineole	Bark	GC-MS	[78]
Camphor	Bark	GC-MS	[78]
endo-Borneol	Bark	GC-MS	[78]
Linalool	Bark	GC-MS	[78]
1-α-Terpineol	Bark	GC-MS	[78]
α-Caryophyllene oxide	Bark	GC-MS	[78]
Nopol	Bark	GC-MS	[78]
2-Pinen-4-one	Bark	GC-MS	[78]
(−)−Bornyl acetate	Bark	GC-MS	[78]
1-Tetradecanol (fatty alcohol)	Bark	GC-MS	[78]
5-Octadecene	Bark	GC-MS	[78]
Hexadecanoic acid methyl ester	Bark	GC-MS	[78]
Palmitic acid	Bark	GC-MS	[78]
17-Pentatriacontene	Bark	GC-MS	[78]
Tritetracontane	Bark	GC-MS	[78]
5β-Pregn-11-ene	Bark	GC-MS	[78]
4-Imidozolidinone	Bark	GC-MS	[78]
Naphthalene	Bark	GC-MS	[78]
Quinoline	Bark	GC-MS	[78]
**Flavonoids**			
Caffeic acid	Leaves	TLC	[69]
Quercetin	Bark, leaves	GC-MC, nuclear magnetic resonance NMR	[46,77]
Quercetin-3-*O*-glucoside	Bark, leaves	GC-MC, NMR	[46,77]
**Phenolic**			
*trans*-Triacontyl-4-hydroxy-3-methoxycinnamate	Bark	NMR	[26]
**Polyol**			
Phytic acid	Bark		[72]
**Sterols**			
Ergosterol	Fruits	GC-MC	[46,77]
5β,6β-Epoxy-7-bromocholestan-3-one	Fruits	GC-MC	[46]
**Tannins**			
Ellagic acid	Bark	TLC	[69]
Gallic acid	Bark	TLC	[69]
**Triterpenes**			
Acacic acid lactone	Bark, leaves	GC-MC, NMR	[46,77]
Betulinic acid	Bark	NMR	[26]
Catechin	Bark	NMR	[26]
Cycloartenol	Fruits	GC-MC	[46,77]
Cycloartenol acetate	Fruits	GC-MC	[46,77]
Epifriedelinol	Bark	TLC	[69]
Friedeline	Bark	NMR, TLC	[26,69]
Oleanolic acid	Bark, leaves	GC-MC, NMR	[46,77]
Taraxerole	Bark	TLC	[69]
Taraxerone	Bark	TLC	[69]
